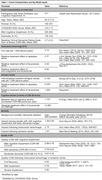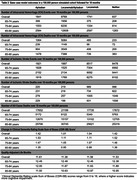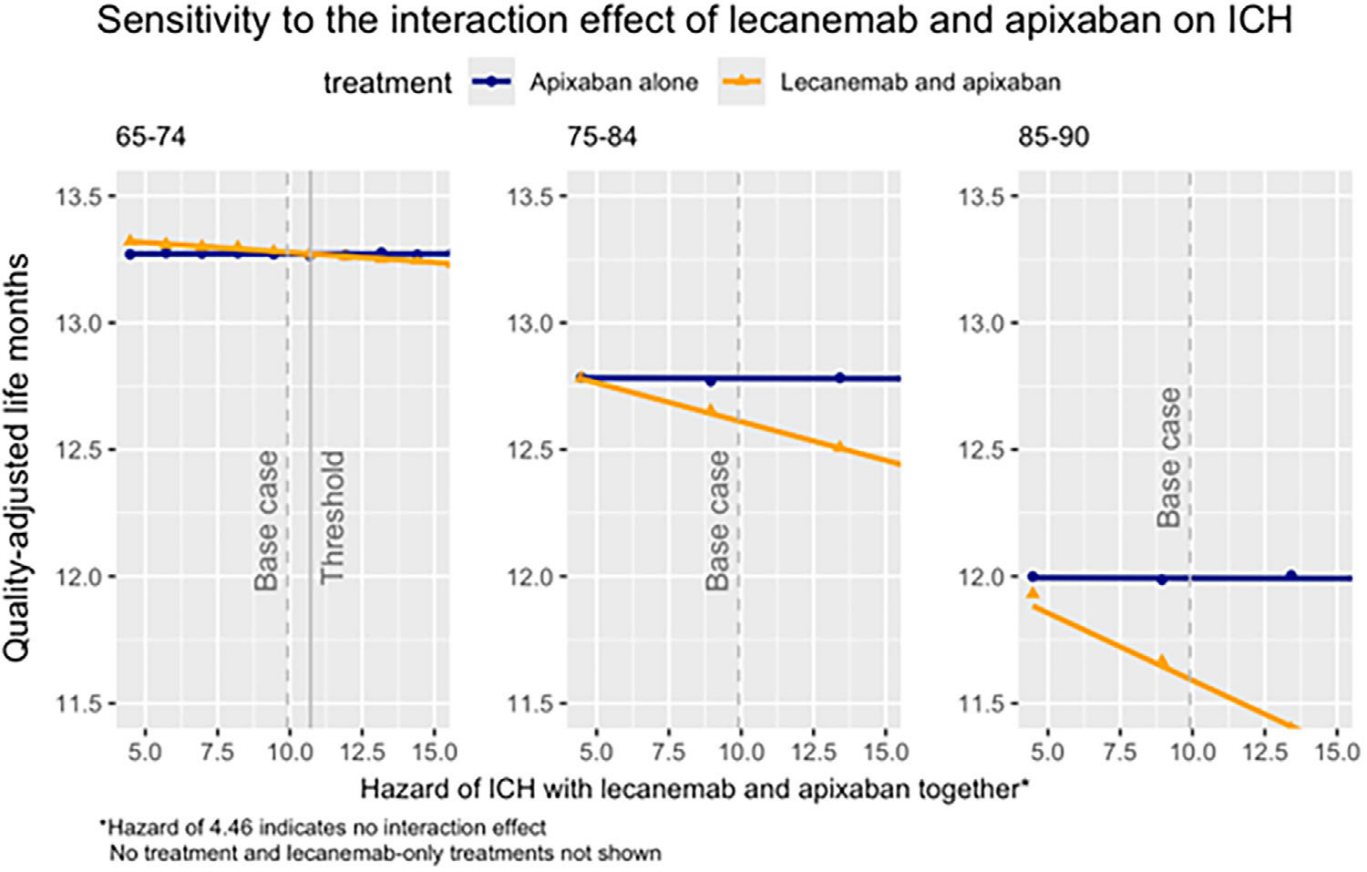# Net Benefit of Lecanemab and Anticoagulants: Results from a Simulation Model

**DOI:** 10.1002/alz.094620

**Published:** 2025-01-09

**Authors:** Sachin J Shah, Tai Dinger, Deborah Blacker, Steven M. Greenberg, Joseph Newhouse, Jacquelyn M Lykken, Ankur Pandya, John Hsu, Emily P Hyle

**Affiliations:** ^1^ Massachusetts General Hospital, Boston, MA USA; ^2^ Harvard Medical School, Boston, MA USA; ^3^ Harvard University, Cambridge, MA USA; ^4^ Department of Epidemiology, Harvard T. H. Chan School of Public Health, Boston, MA USA; ^5^ Harvard T.H. Chan School of Public Health, Cambridge, MA USA

## Abstract

**Background:**

In CLARITY‐AD, lecanemab both slowed cognitive decline and increased intracranial hemorrhages (ICHs), particularly among participants concurrently using anticoagulants. The Alzheimer’s Association’s expert guidance is to avoid co‐prescribing; however, CMS and FDA do not restrict or warn against co‐prescribing. We used a microsimulation model to quantify the potential benefits and harms of co‐prescribing lecanemab and apixaban in people with atrial fibrillation (AF) experiencing mild cognitive impairment or early Alzheimer’s.

**Methods:**

We developed a microsimulation model to estimate the health and cognition‐related quality of life among persons 65‐90 years with AF and cognitive impairment. We compared four strategies over 18 months in a cohort of 100,000 people: apixaban alone, lecanemab and apixaban, lecanemab alone, and neither. We populated the cohort using the national Health and Retirement Study‐AF cohort. Monthly model outcomes included ICH, ischemic stroke, cognitive impairment, quality‐adjusted life months (QALMs), and survival. Increased ICH risk was a key input: a trial‐reported 2.02‐fold increase for lecanemab alone, a 2.21‐fold increase for apixaban alone (anticoagulant literature), and a trial‐reported 9.92‐fold increase for lecanemab and anticoagulants together. We assigned quality‐of‐life estimates and mortality rates for people with cognitive impairment, stroke, and ICH. Background mortality rates increased with cognitive decline and following a stroke or ICH event.

**Results:**

Over 18 months, apixaban alone would result in more QALMs (12.39 vs. 12.16), fewer ICH events (1,841 vs. 8,769), fewer deaths (17,652 vs. 21,199), but more cognitive decline (CDR‐SB increase from baseline 1.42 vs. 1.01) compared to apixaban and lecanemab together (**Table 2**). In the 65 to 74‐year‐old age group, lecanemab and apixaban could be preferred (QALM 13.27 vs. 13.28) given the benefits of slowed cognitive decline but at the risk of 1,424 more ICH events/100,000 treated persons (**Table 2**). This finding is sensitive to the lecanemab‐apixaban interaction on ICH risk (**Figure**).

**Conclusion:**

These model‐based results support apixaban alone as the preferred strategy for people with cognitive impairment and AF. Improved estimates of the lecanemab‐anticoagulant interaction are critical to identifying the preferred strategy for people aged 65‐74 years. These findings support the Alzheimer’s Association’s expert guidance to avoid co‐prescribing lecanemab and anticoagulants.